# Chicago Medical Society

**Published:** 1886-02

**Authors:** 


### CHICAGO MEDICAL SOCIETY.

Officially Reported for The Atlanta Medical and Surgical Journal.

Stated Meeting, January 4, 1885.

The President, C. T. Parkes, M. D., in the chair.

The first paper read was entitled

### THE EFFECTS OF COCAINE ON THE CENTRAL NERVOUS SYSTEM.

*By Dr. D. R. Brower.*

We have recovered from the primary effects of the brilliant discovery of Dr. Carl Koller, that sixteen months ago electrified the medical world, and can now reason together calmly and dispassionately about this powerful therapeutic agent.

I have been using in private and hospital practice the preparation of the coca leaf for about six years, and for about one year past the alkaloid cocaine, and have reached certain conclusions as to its beneficial and its baneful effects on the central nervous system, that I propose to present for discussion in this paper. I say beneficial and baneful effects, for my first proposition is, that it is as powerful for evil as it is for good.

The doctor then in a very able manner elaborated the following facts:

1. Cocaine in small or moderate doses is a cerebral stimulant, but produces derangement of the digestive and assimilative functions, and diminishes the elimination of waste.

2. The use of cocaine in the alcoholic and opium inebriates is not satisfactory; while it is a more or less perfect substitute, yet its use is attended with greater danger than alcohol or opium.

3. The use of cocaine in mental depression, if we carefully guard against the depressing effects of the drug upon digestion and assimilation, will often give better results than any drug hitherto used.

4. The use of cocaine in neurasthenia is a valuable addition to the treatment.

5. The drug, if administered in large doses persistently, causes a very marked deterioration of the central nervous system, producing a profound cerebral neurasthenia, and may produce such a mal-nutrition of the cerebrum as to develop insanity.

6. Cocaine occasionally, in doses heretofore regarded as small, produces alarming depression of the central nervous system.

Dr. E. L. Holmes opened the discussion by saying that the South American Indians ate large quantities of the coca leaf without injury.

Dr. D. R. Brower said that in the early history of Peru the Catholic Church authorities sought to stop the use of coca in that way because of its deleterious effects upon the people. But it was impossible to do it; they used it clandestinely, and the prohibition was withdrawn. Dr. Brower said that, used in that way, the result was a similar condition of mental deterioration as that mentioned in the paper ; while these people were capable, under its influence, of performing muscular efforts in climbing the hills and mountains of that country, they were a puny, sallow, emaciated people, with intellectual capacity very little above the brute creation.

Dr. E. L. Holmes said that his experience had been wholly in connection with local applications on the eye. He had seen one case, in which he had placed cocaine in both eyes for an operation, in which it was followed by considerable depression and nausea through the night, but no alarming symptoms. He had twice used it in his office for strabismus, placing a not unusual quantity in the eye, where the patient felt sick and almost slipped out of the chair, and the operation was performed with the patient lying on the floor, but he suspected it to be a fit of fainting at sight of the instruments. He had found from experience that very minute doses will be absorbed, but had never seen the slightest influence from the amount which is usually given in the eye. It is exceedingly satisfactory in performing operations, and in removing little motes and pieces of iron or steel lodged in the cornea.

Dr. C. W. Earle said it appeared to him that when Dr. B. was in the Washingtonian Home he got along with a very small amount of the drug, very much less than Dr. Earle had anticipated it would be possible for him to get along with, that Dr. B. informed him that he had been in the habit of taking from 15 to 18 grains a day. After he went into the Home, the first three days he took less than grs. iij, and after that time did not take any. He regained his appetite very well, and appeared to improve in every respect up to the time that his wife and brother or brother-in-law came from Canada to visit him; then he was seized with an idea that he must go home, or at least to Canada, and from that time he was uneasy and did not do well. Dr. Earle said that it seemed to him that there had been more said in regard to the use of cocaine than there was any use in saying. While Dr. B. was at the Home he was not unlike an ordinary opium eater. The depression which followed withdrawal was somewhat similar, although there were none of the symptoms of dizziness, or nausea and sneezing, which are usually present after the withdrawal of opium.

Dr. Sarah Hackett Stevenson said that she was surprised at the deleterious effects found by Dr. Brower from the use of cocaine in hay fever. She had had a number of cases in which it was used without any of the symptoms mentioned in the paper. She used a 4 per cent. solution, applying with a camel's-hair brush, or simply by snuffing. Dr. Stevenson said she had used cocaine a great deal hypodermically in surgical operations with good effect so far as anæsthesia was concerned.

Dr. W. F. Coleman said there are two lines of practice in treating the narcotic habit: to cut a man off from his drug completely, at once, or to taper him off. He believed in depriving him of the drug at once. If he found a man being poisoned he would not add another dose, but stop it immediately. He knew nothing practically of the internal use of cocaine, but was surprised at the grave effect mentioned by Dr. Brower. Cohn-Mueller asserts that he kills a frog with four-tenths of a grain, but to human patients he gave indiscriminately five grains and it produced no perceptible ill effect. Locally Dr. Coleman had had some experience with the drug, and there is this point about its immediate effect—it relieves pain in acute otitis, but it is a grave question whether it does not prolong the case, and while it relieves pain it is doubtful if it is better than hot water or other remedies. In its application to the eye there was not this objection; it produces anæsthesia and allows almost any operation to be performed without pain; it does not prolong congestion, and gives permanent relief.

Dr. D. W. Graham said that he noticed that Dr. Brower quoted a case reported by Dr. Burchard in the *Medical Record*, in which a patient sustained loss of consciousness and stoppage of the pulse after an injection of four-fifths of a grain of cocaine. Dr. Graham doubted very much whether the bad effects were due to cocaine; we get the same effect in many patients by injecting water, or by showing them the hypodermic syringe, and he thought it probable that it was simply a fainting fit. There was nothing in the report that would lead to any other conclusion, except that Dr. Burchard says it was the result of the injection of cocaine. Dr. Graham doubted the conclusion and thought it unreliable.

Dr. F. M. Weller said he had had some experience in the use both of cocaine and the fluid extract of coca. He had never used such large doses as those mentioned, but had seen that very large doses of the drug would produce unpleasant symptoms about the head, dull, heavy headache, something like doses of other narcotics. Hypodermically he had used as much as five grains at one time without any unpleasant effects whatever. He was inclined to think that the real difficulty in some of these cases was that some other circumstance was overlooked. It seemed to him that some idiosyncrasy might exist that would make one peculiarly susceptible to this kind of narcotic, and it would hardly be reasonable to charge it all to cocaine. His reading of the history of the use of the drug in South America, where it originated, led him to think that it could be used a long time without injury. He thought one lesson is to be learned from the paper, viz., that no drug should be continued beyond the time of its necessity. That principle laid down and strictly adhered to, the patient would never suffer from the deleterious use of any drug.

Dr. D. R. Brower, in closing the discussion, said that Dr. B. bought cocaine wherever he could get it, and he did not know what preparation he used. The other physician mentioned in the paper used Merck's cocaine altogether. He used it for hay fever and took five grains by inhalation every day for ten days. Dr. Brower said that he did not suppose that such disastrous effects as occurred in these cases and others would result unless there was some weakness of the nervous system; he thought possibly a person perfectly robust and with a well-governed nervous organization could take cocaine with impunity, but a person with a nervous temperament could not use this drug continuously without some such results following. Dr. Brower said his plan of treatment was that of gradual withdrawal, as well with opium and chloral as with cocaine. This plan could be followed with less inconvenience to the patient. Dr. Brower said he was well aware that cocaine was one of the most valuable additions to the therapeutics of hay fever; it had been administered in perhaps thousands of cases in Chicago, and with few disastrous effects.

As to the case reported by Dr. Burchard, the Doctor reported it as a case resulting from hypodermic injection, and it struck Dr. Brower as being a reasonable result in a very susceptible individual, but it might be that the man had a fainting fit. The case was reported in the *Medical Record* of December 5, 1885; it impressed Dr. Brower as being undoubtedly a case of cocaine poisoning.

After the reading of the paper entitled "Opium Smoking," by Dr. C. W. Earle, and one entitled "Hygienic Clothing," by Dr. L. L. McArthur, on which there was no discussion, the Society adjourned.

---

FRIEDRICHSHALL WATER IN THE TREATMENT OF HABITUAL CONSTIPATION.—"The mildness of the effect of Friedrichsh[illegible] water in these cases is, according to my own observations, w[illegible] marked. In no case, even after prolonged use, did it prove in any way excessive, and, when its use is abandoned for a time, the constipation only recurs gradually when its regulating action on the intestines has given place to the habits of the body or errors in diet which produce the tendency to constipation in the first intance. The non-occurrence of the reactionary constipation which so generally follows the habitual use of aperients is doubtless to be attributed to the large quantity of chlorides present in Friedrichshall water, and to their favorable inflence on the progress of digestion and diffusion. In short, the class of cases in which I have found this water of especial service comprises hemorrhoids, accompanied by habitual constipation, hepatic congestion, and the constipation of pregnancy, which is so often complicated by derangements of the digestion, headache and dyspnœa. There are many cases of cardiac disease and of cardiac irritability in young people in which it is very serviceable in continuous and diminishing doses. It has long had a high reputation in the treatment of gravel and for the prophylactic treatment of renal calculi, but of this I am unable to speak from experience. It has appeared to produce a favorable impression in the numerous cases of strumous and glandular swellings, where we have to contend with a sluggishness of the bowels and of tissue change generally. Here Friedrichshall water acts as a stimulant as well as an aperient. A certain diuretic effect which is manifested is not uncommonly of service in cases where it is desired to increase the proportion of watery constituents of the urine."—*Mr. A. S. Gubb, Medical Press and Circular, Nov. 11, 1885.*

